# Penetration and Cratering of Steel Target by Jets from Titanium Alloy Shaped Charge Liners

**DOI:** 10.3390/ma15145000

**Published:** 2022-07-18

**Authors:** Dacheng Gao, Wenbin Li, Wenjin Yao, Kebin Zhang, Yiming Li, Ping Song, Bo Pu

**Affiliations:** 1 Ministerial Key Laboratory of ZNDY, Nanjing University of Science and Technology, Nanjing 210094, China; dc001@njust.edu.cn (D.G.); njyaowj@njust.edu.cn (W.Y.); kb2018@njust.edu.cn (K.Z.); 316101002454@njust.edu.cn (Y.L.); 2 Research Institute of Chemical Defense, Beijing 102205, China; sp2016@njust.edu.cn; 3 Beijing Special Electrical and Mechanical Research Institute, Beijing 102205, China; 2022dc@njust.edu.cn

**Keywords:** titanium alloy, shaped charge, penetration, crater radius

## Abstract

In order to enlarge the crater diameter of shaped charge jet penetration into steel targets, this paper investigates the penetration and cratering characteristics of steel targets by shaped charge jets from titanium alloy liners. Titanium alloy shaped charge liners are prepared separately with mechanical processing and selective laser melting (SLM), and pulsed X-ray radiography is used to identify jet formation characteristics. Jet formation is numerically simulated by AUTODYN-3D, and steel target penetration tests are carried out at a short jet stand-off distance. The results show that AUTODYN-3D can realistically simulate jet formation from titanium alloy liners and that the SLM-processed liner exhibits better penetration performance than the mechanically processed liner. The existing cratering formula of jet penetration is modified to make it consistent with the aperture variations of jet penetration from titanium alloy-lined shaped charges at a short stand-off distance. The findings of this study are expected to provide technical and theoretical support for research on the penetration characteristics of the jets from titanium alloy-lined shaped charges.

## 1. Introduction

Liner material properties greatly affect jet penetration and cratering and even the fulfillment of operational tasks as a result [1]. Liners made of high-density materials such as copper and tantalum generally deliver a high penetration depth into steel targets, but the crater diameter is relatively small. Conversely, liners made of low-density materials such as aluminum and magnesium exhibit low penetration power but good cratering performance [2,3]. With the advantages of uniform composition, small grain size, high specific strength, and high corrosion resistance, titanium alloys enjoy promising application prospects for liners [4,5].

In recent years, some research efforts have been made on titanium alloy shaped charge liners. With a combination of experimental and numerical simulations, Kang et al. [6] investigated the penetration differences of jets from titanium alloy, low-carbon steel, and red copper shaped charge liners into a multi-layer target. Zhang et al. [7] experimentally studied the penetration of jets from two types of large-cone-angle titanium alloy liner and found that the jets from the titanium alloy liners produced a much larger crater diameter than the jet from copper liners. E et al. [8] designed a high-density titanium alloy shaped charge liner with a 120° cone angle and obtained a crater on a steel target after jet penetration. Hao et al. [9] utilized titanium alloy-lined shaped charge jets to penetrate pure copper and carbon steel targets and concluded that the penetration of titanium alloy-lined shaped charge jets into carbon steel targets would lead to violent chemical reactions and severe lateral dissipation. Yan et al. [10] introduced direct laser deposition technology into the preparation of TC4 matrix composite-based shaped charge liners so as to improve their penetration performance.

Tandem warheads are an effective weapon for hitting hard targets. The first stage of tandem warheads is a shaped charge, and the depth and diameter of the crater produced by the shaped charge jet need to meet certain conditions [11]. Research has been carried out on penetration depth and targets’ radial response to jet penetration. The representative work in this regard was carried out by Szendrei et al. [12], who developed a crater growth equation. Held et al. [13] modified the Szendrei equation and conducted experimental research. Xiao et al. [14,15,16] derived the relationship between crater growth speed and crater diameter and the cratering formula of jets penetrating concrete and carried out programming calculation and experimental verification. Based on the theoretical analysis performed by Szendrei T and the concept of virtual origin, Wang et al. [17,18] produced the variation law of the crater diameter produced by jet penetration with penetration depth and verified the good agreement between theoretical and experimental results.

The most prominent penetration characteristic of shaped charge jets from a titanium alloy liner is a large crater diameter. At present, there is much research on the penetration of titanium alloy shaped charge jets into concrete but few on the penetration of titanium alloy shaped charge jets into metal targets. With the change in combat mission, it is more necessary to study the penetration and cratering of steel targets by jets from titanium alloy shaped charge liners. Therefore, in this paper, shaped charge liners made of TC4 matrix composites were produced separately by the two techniques of mechanical processing and SLM. With pulsed X-ray radiography, jet morphology and parameters such as jet head velocity and radius were obtained. Numerical simulations were conducted to identify the distribution of jet velocities and the position of the virtual origin. The jet velocity distribution characteristics given by X-ray test results and jet formation numerical simulation were used to modify the radius and velocity of the jet in the existing theoretical model. The modified model is expected to provide technical and theoretical support for research on the penetration characteristics of jets from titanium alloy-lined shaped charges.

## 2. X-ray Testing for Jets from Titanium Alloy Liners

### 2.1. Experimental Design

**Figure 1:**
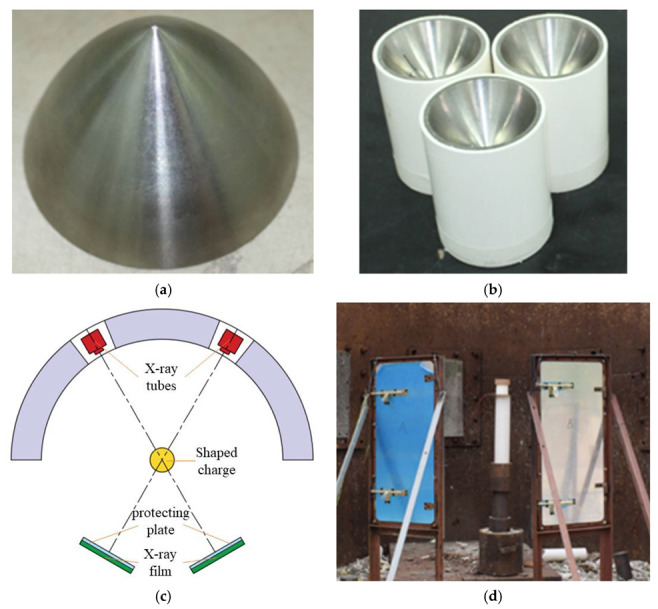
Experimental principle and setup of pulse X-ray. (**a**) Titanium alloy liner; (**b**) shaped charge structure; (**c**) schematic; (**d**) test setup.

The titanium alloy liner was an eccentric sub-hemisphere with variable wall thickness, as shown in Figure 1a. The charge diameter and height of the test projectile were 89 mm and 105 mm, respectively, and 8701 explosive was used as the charge. Figure 1b,c shows the real shaped charge and the test layout. The shaped charge was detonated at the midpoint. The test system consisted of two X-ray tubes intersecting at 45°, a test projectile, a height of burst (HOB) drum (PVC tube), two film holders, and four films. The test projectile was placed on the HOB drum, and the vertical HOB drum was ensured at the intersection of the two X-ray tubes. The light-emitting time of the X-ray machine was set so that two X-ray images at different times could be obtained at once. Before the test, numerical simulations were carried out to adjust the placed height of the test projectile and the light-emitting time of the X-ray machine. Each film holder contained two films to ensure that jet formation could be completely presented on the films. Upon the detonation of the charge, air ionization would make the originally disconnected wire connected, which would serve as the trigger signal of the X-ray machine.

### 2.2. X-ray Test Results

The post-test X-ray films were scanned into pictures, which were treated by software to make the jet thereon more clearly visible. The results are shown in Table 1. If the jet is present on two sequential films, they should be spliced into one. XT-1 and XT-2 were the liners produced by mechanical processing and SLM, respectively. The XT-1 image at the time of *t* = 76 μs was made by splicing two X-ray films. For the XT-2-induced jet, the rod was not completely shown on the X-ray images at the two moments, and so the jet length could not be measured accurately.

As can be seen from Table 1, the jets of the same moment formed by the two types of titanium alloy liner showed no difference in shape, and both underwent dispersion during the stretching process. That is, the jets no longer remained integral but appeared in a particle-like state. In addition, with the diameters of the liner mouth and HOB drum as the scale, jet head and tail positions at different moments could be measured so as to obtain the jet head and tail average velocities and jet head diameter. The main data of the measured jet are shown in Table 2. The jet from the SLM-processed liner exhibited a higher head velocity and a larger head radius, suggesting that the SLM-processed liner has some advantages.

## 3. Numerical Simulation of Jet Formation from Titanium Alloy Liners

### 3.1. Numerical Calculation Model and Material Parameters

Considering jet dispersion in the stretching process, jet formation was numerically simulated by the smooth particle hydrodynamics (SPH) method using AUTODYN-3D. SPH [19,20] is a mesh-free particle method based on Lagrangian formulation which describes material deformation through the motion of particles rather than the mesh. The interaction between materials can be naturally simulated by particle interactions, which can effectively avoid such problems as mesh distortion and negative volumes in the finite element method. Therefore, the SPH method is widely used in fields such as ultra-high-speed impact, explosion, crack propagation, and metal forming [21,22,23].

**Figure 2:**
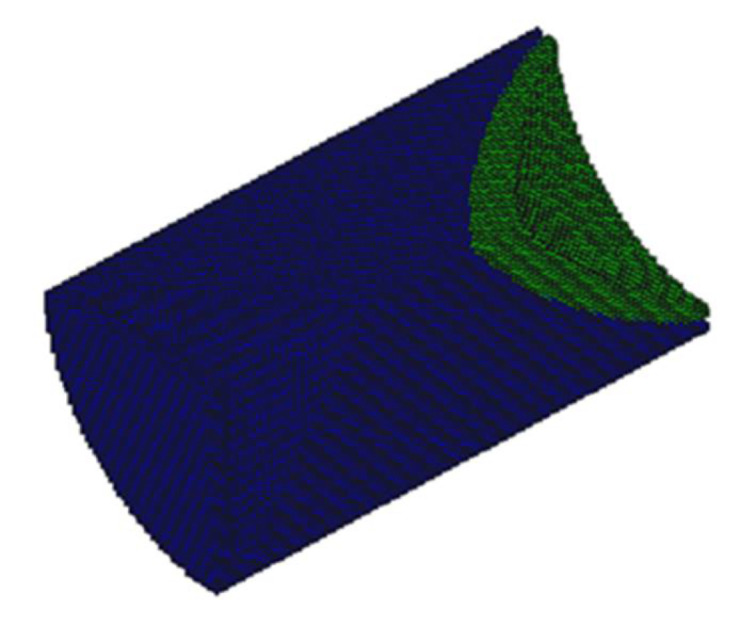
The quarter model for shaped charge numerical calculation.

According to the symmetry of the shaped charge structure, a quarter model was built for the sake of calculation, as shown in Figure 2.

For the SPH method, particle spacing is a parameter of great importance. The particle spacings were set to 0.8 mm, 1.0 mm, 1.2 mm, and 1.5 mm, separately. When it was 1.0 mm, the X-ray test results could nearly be obtained. In the numerical calculation, the Johnson–Cook (J–C) strength model and shock equation of state (EOS) were used to describe the TC4 titanium alloy. The J–C model represents the strength behavior of typical metals subjected to large strain, high strain rate, and high temperature. The model comprehensively considers the impacts of strain rate effects and temperature on flow stress. In [24], based on the experimental data, the J–C constitutive model of SLM-processed TC4 alloy was established. The J–C constitutive model established in [24] could better describe the plastic flow behavior of the TC4 alloy under high-temperature and high-strain-rate deformation conditions. Here, the model parameters in [24] were employed, but the density parameter was modified into 4314 kg/m^3^, i.e., the average density of the two types of liner. For the shock EOS, the parameters in the software’s in-built material library were utilized. The 8701 explosive was used as the charge. The JWL EOS was adopted to describe the detonation and energy release processes of the explosive. The density was 1690 kg/m^3^; the detonation velocity was 8425 m/s; and the C–J pressure was 29.5 GPa. The detailed parameters of the 8701 explosive are shown in Table 3. The pressure is expressed as:

(1) $$p_{E}=A\left(1-\frac{\omega }{R_{1}V}\right)\exp\left(-R_{1}V\right)+B\left(1-\frac{\omega }{R_{2}V}\right)\exp\left(-R_{2}V\right)+\frac{{\omega E}_{0}}{V}$$

where *p*_E_ is the detonation pressure; *V* = 1/*ρ* is the relative volume; *ρ* is the density of the explosive; *E*_0_ is the specific internal energy per unit of mass; and *A*, *B*, *R*_1_, *R*_2_, and *ω* are the material constants.

### 3.2. Comparison of Numerical Simulation and X-ray Testing Results

**Figure 3:**
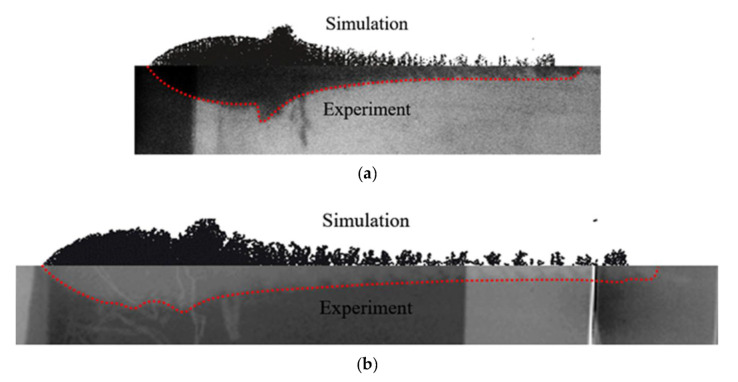
Comparison of numerical simulation and test results over jet formation; (**a**) *t* = 56 μs; (**b**) *t* = 76 μs.

Figure 3 and Table 4 show the comparison of the numerical simulation and X-ray testing results of the shaped charge jet at 56 μs and 76 μs.

It can be seen from Figure 3 and Table 4 that the numerical simulation results were consistent with the experimental results, indicating relative consistency in the formation process and providing effective jet velocity distribution information. What is more, the shaped charge jet formed by the titanium alloy SLM-processed liner exhibited a higher head velocity. The results of the numerical simulation are ideal, but there were many uncertainties in the test process. The defect of the charge, the machining error of the titanium alloy liner, and the position error of the detonator lead to deviation of the test results. The error between the numerical simulation and the experiment is acceptable if it is within a certain range. Therefore, the numerical simulation results can provide a basis for subsequent theoretical calculation.

## 4. Jet Penetration Test from Titanium Alloy Liners into Steel Target

**Figure 4:**
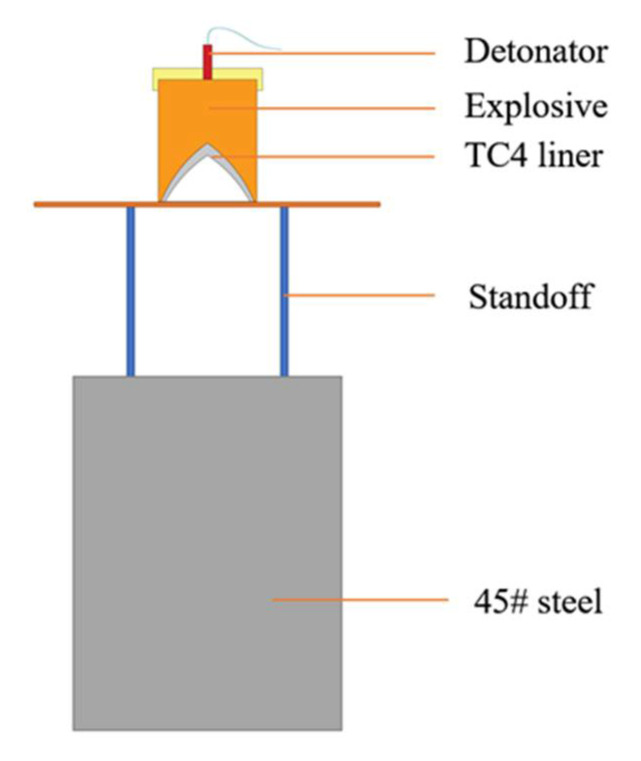
Schematic diagram of jet penetration into steel target.

**Figure 5:**
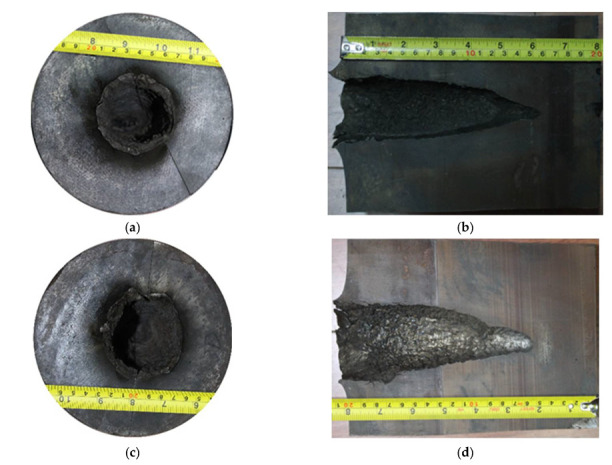
Steel target penetration results; (**a**) top view: PX-1; (**b**) section view: PX-1; (**c**) top view: PX-2; (**d**) section view: PX-2.

The X-ray test shows that the jets from the two types of liner appear discrete during the stretching process. Since jet breakup goes against penetration, for the sake of mitigating its adverse effects, the stand-off (distance between the bottom of the shaped charge and the surface of the target) was set to double the radius of the charge in the test, i.e., 89 mm, lower than the normal burst height of shaped charge jets. The steel target was made of C45 steel with a diameter of 150 mm and a height of 200 mm. The test arrangement is shown in Figure 4. After the test, the steel target was cut open to understand the aperture diameter variations with penetration depth. The penetration results are shown in Figure 5 and Table 5, where PT-1 and PT-2 are the mechanically and SLM-processed liners, respectively.

As shown in Table 5, the jet from the SLM-processed liner performed better in both inlet radius and penetration depth. Titanium alloys can have a violent combustion reaction with N_2_ in the air at high temperatures [25], and, moreover, both Ti and Al in titanium alloys can react violently with Fe. The surface of the penetration channel is magnified for observation, and obvious traces of high-temperature erosion are seen. In addition, the surface of the channel is rough, with many cracks and silver-white residual jets visible to the naked eye.

## 5. Variation Laws of Penetration Depth and Aperture Diameter

The most prominent penetration characteristic of shaped charge jets from a titanium alloy liner is a large crater diameter, and the penetration depth of such jets is also the focus of research. Therefore, based on the jet penetration and cratering model in [18], this study explored the cratering characteristics of shaped charge jet penetration from titanium alloy liners into steel targets at a short stand-off distance. Below is the theoretical calculation of the penetration depth and aperture diameter.

According to the virtual origin assumption developed by Allison and Vitali [26], the jet penetration depth at time t can be expressed as:

(2) $${P(t)=v}_{j}{(t)t-z}_{0}$$

where *P*(*t*) is the penetration depth; *v*_j_(*t*) is the velocity of the jet unit penetrating the target plate; and *z*_0_ is the distance from the virtual origin to the target surface.

Combining with Bernoulli’s equation, Equation (2) is differentiated and then integrated:

(3) $$v_{j}\left(t\right){=v}_{0}{\left(\frac{t_{0}}{t}\right)}^{\frac{\gamma }{1+\gamma }}$$

where *γ* = (*ρ*_t_/*ρ*_j_)^0.5^, in which *ρ*_t_ and *ρ*_j_ are the densities of the target and the jet, respectively; $v_{0}$ is the velocity of the jet head when it touches the target; and *t*_0_ is the moment when the jet head touches the target, i.e., *v*_0_*t*_0_ = *z*_0_.

After Equation (3) is substituted into Equation (2), the penetration equation can be expressed as:

(4) $$P\left(t\right){=z}_{0}\left[{\left(\frac{t_{c}}{t_{0}}\right)}^{\frac{1}{1+\gamma }}-1\right]{=z}_{0}\left[{\left(\frac{v_{0}}{v_{j}}\right)}^{\frac{1}{\gamma }}-1\right]$$

Assuming that the penetration stops at time $t_{c}$ and the terminal infinitesimal velocity of the jet is $v_{c}$, the maximum penetration depth *P*_max_ can be given by

(5) $$P_{\max}{=z}_{0}\left[{\left(\frac{t_{c}}{t_{0}}\right)}^{\frac{1}{1+\gamma }}-1\right]{=z}_{0}\left[{\left(\frac{v_{0}}{v_{c}}\right)}^{\frac{1}{\gamma }}-1\right]$$

Equation (5) is the penetration formula for jets from a shaped charge liner made of single materials, represented by copper, and so it is only suitable for jets from constant-density liners. The virtual origin model retains the basic equations in the hydrodynamic model, where the strengths and the compressibility of the jet and target materials are neglected, but considers the jet stretching as it travels forwards. This model has been widely accepted and can be used to predict the penetration depth before and after jet breakup [27,28,29,30].

**Figure 6:**
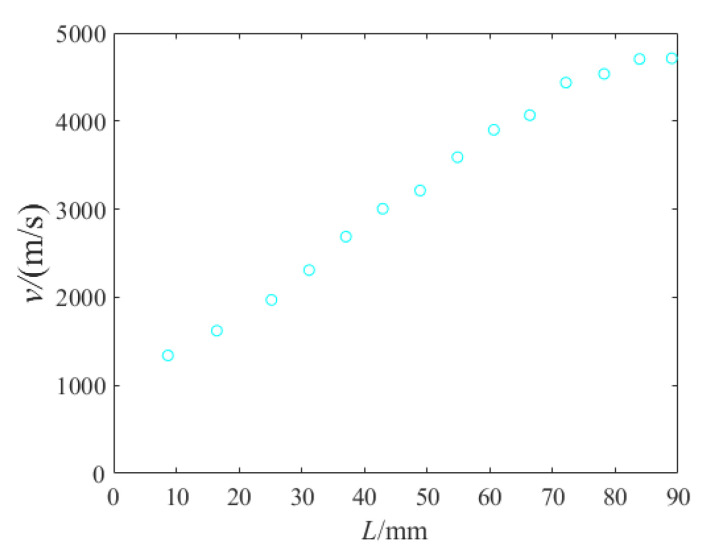
Jet velocity distribution.

The velocity distribution obtained through the jet formation numerical simulation is shown in Figure 6. The time is the moment when the jet head reaches the steel target, and *L* represents the distance from the jet to the bottom of the liner. With the least squares method, the distance from the virtual origin to the target can be worked out; *z*_0_ = 90.5 mm.

The penetration depths and head velocities of PT-1 and PT-2 were substituted into Equation (5) to fix the position of the virtual origin through the aforesaid numerical simulation. The velocities $v_{c}$ of PT-1 and PT-2 were calculated, namely, 1275 m/s and 1300 m/s. It can be seen that the two values were approximate, suggesting that the two tests are highly consistent.

Based on the concept of virtual origin, Wang et al. [17,18] gave the variation law of crater diameter with penetration depth:

(6) $$R_{f}=r{\left[\frac{9}{4}+\frac{1}{1+\frac{\sigma }{\rho_{t}{\left(\frac{v}{1+\gamma }\right)}^{2}}}\right]}^{0.5}{\left[1+\frac{1}{4\frac{\sigma }{\rho_{t}{\left(\frac{v}{1+\gamma }\right)}^{2}}}\right]}^{0.5}$$

where *R*_f_ is the cavity radius; σ is the parameter of target strength; γ = √(*ρ*_t_/*ρ*_j_), in which *ρ*_t_ and *ρ*_j_ are the densities of the target and the jet; *r* = *r*_0_ (*s*/(*P* + *s*))^0.5^ and represents the head radius of the jet when the penetration depth is *P*, in which *r*_0_ is the head radius of the jet at the time of landing; *v* = *v*_0_(*s*/(*P* + *s*))*^γ^* and represents the velocity of the jet when the penetration depth is *P*, in which *v* is the head velocity of the jet at the time of landing; and *s* is the distance from the virtual origin to the target, i.e., *z*_0_.

**Figure 7:**
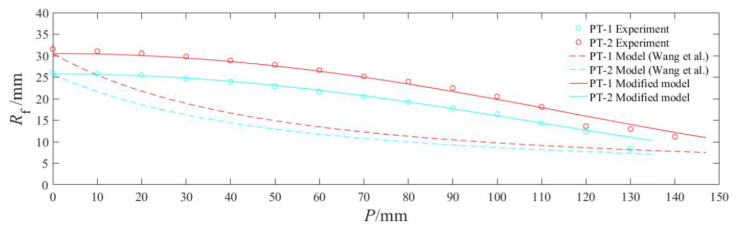
Comparison of the experimental data with the model in [18] and the modified model.

Except for the target strength *σ*, all parameters can be obtained from X-ray radiography and numerical simulation. In the numerical simulation, *s* = 90.5 mm was obtained by the least squares method. The strength parameter *σ* can be calculated inversely using the aperture formula, i.e., *σ* = 1000 MPa according to the inlet radius of PT-1 and the jet head velocity and radius of XT-1. The variation curves of PT-1 and PT-2′s apertures with the penetration depth were drawn. In addition, the aperture of the steel target was measured every 10 mm so that a scatter diagram was drawn, as shown in Figure 7.

As illustrated, the theoretical inlet radii of PT-1 and PT-2 were in good agreement with the experimental results, suggesting the validity of the inversely calculated target strength value. Nonetheless, the aperture exhibited an inconsistent variation trend over penetration depth, and so Equation (6) needs to be modified to conform to the variation law of the aperture produced by jets from a titanium alloy liner penetrating steel targets at a short stand-off distance. The observation and measurement results of X-ray images revealed that the radius of the jet did not change significantly during the stretching process, which disagrees with the reducing *r* in the original theoretical formula. In view of this, it is desirable that *r* = *r*_0_. In addition, according to the jet velocity distribution in Figure 6, the velocity in the original theory was modified. It was assumed that the velocity change conformed to the cosine function, so *v* in Equation (6) was modified as *v* = *v*_0_cos(*γP*/*P*_max_).

Thus, Equation (6) can be rewritten into:

(7) $$R_{f}{=r}_{0}{\left[\frac{9}{4}+\frac{1}{1+\frac{\sigma }{\rho_{t}{\left(\frac{v_{0}\cos\left({\gamma P/P}_{\max}\right)}{1+\gamma }\right)}^{2}}}\right]}^{0.5}{\left[1+\frac{1}{4\frac{\sigma }{\rho_{t}{\left(\frac{v_{0}\cos\left({\gamma P/P}_{\max}\right)}{1+\gamma }\right)}^{2}}}\right]}^{0.5}$$

where *P*_max_ is the maximum penetration depth. The modified aperture change curve was drawn and then compared with the model in [18] and experimental curves, and the results are shown in Figure 7. As illustrated, the modified and experimental apertures changed consistently, except with a slightly larger error at the end.

## 6. Conclusions

The jets from the titanium alloy shaped charge liners designed in this paper produced a large crater in the wake of the penetration into the steel target at a short stand-off distance. The jet from the SLM-processed liner exhibited a higher head velocity, which is an important factor affecting the aperture diameter and penetration depth. Specifically, the SLM-processed liner resulted in an aperture diameter 0.709 times the charge diameter, 20% larger than that made by the mechanically processed liner, which was 0.585 times the charge diameter. Moreover, the SLM-processed liner led to a 10% higher penetration depth. It is feasible to perform X-ray tests for comparison and to numerically simulate jet formation with the SPH method using AUTODYN-3D. The numerical simulation results were consistent with the experimental results, indicating relative consistency in the jet formation process and providing effective jet velocity distribution information. Therefore, the numerical simulation results can provide a basis for theoretical calculation.

The penetration of jets from titanium alloy liners into steel targets at a short stand-off distance resulted in an aperture changing differently from that produced by conventional jets. Therefore, there was a large error between theoretical and experimental results in aperture variations with penetration depth. The observation and measurement results of X-ray images revealed that the radius of the jet did not change significantly during the stretching process. In view of this, the radius of the jet in the original theory was modified. According to the jet velocity distribution, it was assumed that the velocity change conformed to the cosine function; the velocity in the original theory was modified. The modified model conforms to the variation law of the aperture produced by jets from a titanium alloy liner penetrating steel targets at a short stand-off distance. The results from the modified model were found to be well consistent with the experimental data. However, the modified theoretical model requires verification by more experiments, and so it remains uncertain whether it is applicable to the aperture variations of jet penetration from other shaped charge structures or liners made of other materials into steel targets at a short stand-off distance.

## Figures and Tables

**Table 1:** X-ray test results.

Liner	*t* = 56 μs	*t* = 76 μs
XT-1	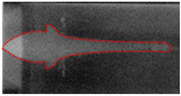	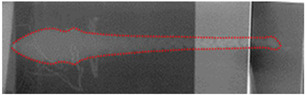
XT-2	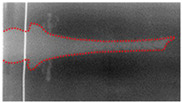	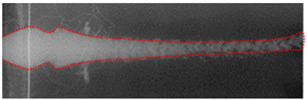

**Table 2:** The main data of the measured jet.

Result	Head Velocity (m/s)	Tail Velocity (m/s)	Head Radius (mm)	Liner Density/(kg/m^3^)
XT-1	4850	1350	5.19	4459
XT-2	4320	1215	5.60	4200

**Table 3:** 8701 explosive parameters.

*ρ* (kg/m^3^)	*D* (m/s)	*P*_CJ_ (GPa)	*A* (GPa)	*B* (GPa)	*R*_1_	*R*_2_	*ω*	*E*_0_ (J/kg)
1690	8425	29.5	8.524	0.18	4.6	1.3	0.38	6.04 × 10^6^

**Table 4:** Comparison of jet parameter numerical simulation and X-ray test.

Results	Head Velocity (m/s)	Tail Velocity (m/s)	Head Radius (mm)	Jet Length at 56 μs (mm)	Jet Length at 76 μs (mm)
XT-1	4850	1350	5.19	174.5	243.4
XT-2	4320	1215	5.60	>163	>244.5
Numerical simulation	4710	1075	5.40	165.9	234.6
Error/%	8.8	11.3	6.0	4.9	3.6

**Table 5:** Penetration test results.

Liner	Initial Crater Radius (mm)	Penetration Depth (mm)	Liner Density (kg/m^3^)
PT-1	26.04	135.1	4428
PT-2	31.54	147.8	4200

## Data Availability

The experimental and numerical modeling results are available upon request.
